# Chemical approaches targeting the hurdles of hepatocyte transplantation: mechanisms, applications, and advances

**DOI:** 10.3389/fcell.2024.1480226

**Published:** 2024-10-31

**Authors:** Huanxiao Shi, Yi Ding, Pingxin Sun, Zhuman Lv, Chunyan Wang, Haoxin Ma, Junyu Lu, Bing Yu, Wenlin Li, Chao Wang

**Affiliations:** ^1^ Department of Cell Biology, Naval Medical University, Shanghai, China; ^2^ Experimental Teaching Center, Naval Medical University, Shanghai, China; ^3^ Shanghai Key Laboratory of Cell Engineering, Naval Medical University, Shanghai, China

**Keywords:** hepatocyte transplantation, small molecule, cell-based therapy, induced pluripotent stem cell, instant blood-mediated inflammatory reaction

## Abstract

Hepatocyte transplantation (HTx) has been a novel cell-based therapy for severe liver diseases, as the donor livers for orthotopic liver transplantation are of great shortage. However, HTx has been confronted with two main hurdles: limited high-quality hepatocyte sources and low cell engraftment and repopulation rate. To cope with, researchers have investigated on various strategies, including small molecule drugs with unique advantages. Small molecules are promising chemical tools to modulate cell fate and function for generating high quality hepatocyte sources. In addition, endothelial barrier, immune responses, and low proliferative efficiency of donor hepatocytes mainly contributes to low cell engraftment and repopulation rate. Interfering these biological processes with small molecules is beneficial for improving cell engraftment and repopulation. In this review, we will discuss the applications and advances of small molecules in modulating cell differentiation and reprogramming for hepatocyte resources and in improving cell engraftment and repopulation as well as its underlying mechanisms.

## Highlights

1. Small molecule modulation of signaling pathways (e.g., Wnt, TGF-beta, HGF) and epigenetic profiles can promote the differentiation of stem cells into hepatocyte-like cells or direct reprogramming of fibroblasts into hepatocytes.
2. Small molecule can maintain hepatocyte function and hepatic progenitor cells self-renewal and expansion.
3. Small molecules can improve hepatocyte engraftment by disrupting endothelial integrity, preventing microcirculatory obstruction, and inhibiting instant blood-mediated inflammatory reaction.
4. Small molecules can establish proliferation advantage of transplanted hepatocytes to enhance liver repopulation.

## 1 Introduction

Huge amounts of patients are being inflicted on liver diseases in the world. Orthotopic liver transplantation (OLT) is the standard treatment for end-stage liver diseases, acute liver failure (ALF), and inherited metabolic liver diseases (Lucey et al., 2023), but many patients die waiting due to the donor scarcity. Hepatocyte transplantation (HTx) is a promising alternative as it can directly restore liver function or bridge patients to OLT (Nulty et al., 2024; Peng et al., 2021). Advantages over OLT include less invasiveness, wider donor sources, repeat injections, and modifiability. Originally proposed in 1976 as a new treatment for Crigler-Najjar syndrome type 1 and validated in hyperbilirubinemic Gunn rat models, HTx has undergone substantial advances over time (Matas et al., 1976). Carrying this work into clinical application, the first human HTx procedure was performed in 1992 utilizing autologous hepatocytes for individuals with cirrhotic liver (Mito et al., 1992). Over the last 3 decades, more than 150 clinical trials of HTx have been reported and manifested efficacy (Gramignoli et al., 2015). Clinical indications for HTx include liver-based metabolic diseases, ALF, and chronic liver failure. In liver-based metabolic diseases, HTx initially improves biochemical indicators and clinical symptoms, but sustained responses are limited, with orthotopic liver transplantation (OLT) typically required after 9–12 months (Nguyen et al., 2020). For instance, Meyburg J et al. reported 4–13 months of metabolic stabilization in four children with urea cycle disorder following primary human hepatocyte (PHH) transplantation. (Meyburg et al., 2009). HTx has shown efficacy in ALF, albeit with variable outcomes. In a clinical trial of seven patients with acute-on-chronic liver failure, three fully recovered, one survived and later underwent OLT, and three died within 2.5–12 months post-transplantation (Wang et al., 2014). Notably, transplanted hepatocytes were detected by MRI in the spleen of two long-term survivors at 48 months post-transplantation (Wang et al., 2014). A recent trial using alginate-coated human hepatocyte microbeads in eight children with ALF demonstrated full recovery in four patients and successful bridging to OLT in three (Dhawan et al., 2020). For chronic liver failure, a phase I-II matched case-control trial of intrasplenic HTx using fetal hepatocytes showed improvements in Child-Pugh scores and encephalopathy compared to the control group, with stable clinical scores and absence of encephalopathy at 1-year follow-up (Pietrosi et al., 2015).

Despite its promising potential, limited high-quality donor hepatocytes and poor engraftment and repopulation hinder large-scale clinical use. PHHs have been widely used in clinical trials which easily lose functions during cell isolation, culture, and cryopreservation (Sun et al., 2023). Hepatocyte-like cells (HLCs) induced from stem cells by growth factors, small molecules, and/or transcription factor transduction are less functional than PHHs(Gao et al., 2020). Low cell engraftment is common in HTx due to instant blood-mediated inflammatory reaction (IBMIR) and chronic immune rejection (reviewed by Sun et al.). The cell engraftment was only 0.1%–0.3% in mice receiving 1 cell infusion of 3%–5% of the total recipient liver cells (Wang et al., 2002). Lack of proliferation and quantity of transplanted hepatocytes contributes to poor liver repopulation.

**FIGURE 1:**
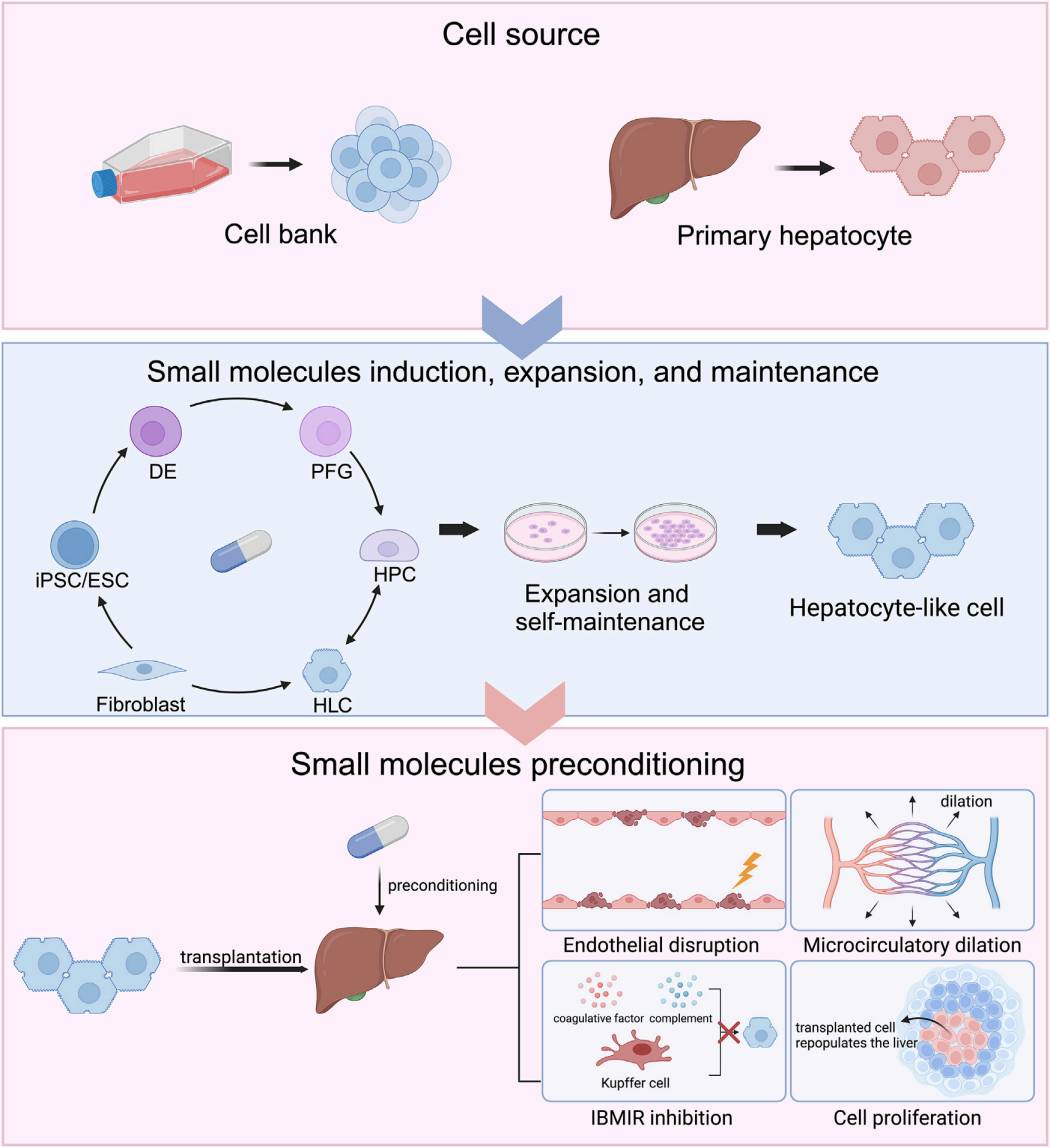
Chemical approaches to generate hepatocytes for cell transplantation and improve cell engraftment and repopulation.

To improve hepatocyte engraftment, preconditioning strategies including irradiation and partial hepatectomy have been established but chemical intervention provide a less invasive approach. Small molecules are widely used tools in stem cell research and manipulating biology through protein interactions (Li et al., 2013; Wang et al., 2023). Compared to genetic techniques, small molecules are convenient to use, concentration-dependent, rapidly reversible, and spatially controlled. This review discusses mechanisms and applications of small molecules in generating high-quality hepatocytes via differentiation and reprogramming and in improving hepatocyte engraftment and repopulation (Figure 1).

Chemical approaches are applied in modulating cell identity in cell differentiation and reprogramming. Primary human hepatocytes (PHHs) are currently used in clinical hepatocyte transplantation (HTx) whereas their loss of function affects HTx efficiency. Small molecule cocktails are established to alleviate the loss of function and promote hepatocyte proliferation. Chemically induced hepatocyte-like cells (HLCs) are another hepatocyte source for HTx. induced pluripotent stem cells (iPSCs) or embryonic stem cells (ESCs) can be sequentially differentiated into definite endoderm (DE), posterior foregut (PFG), hepatic progenitor cells (HPCs), and HLCs under small molecule induction. Hepatocytes can convert to HPCs and thereby expand. HPCs can remain their ability of proliferation and self-maintenance and act as hepatocyte pool by small molecule modulations. Fibroblasts are able to be directly reprogrammed into HLCs under chemical modulation. The host liver can be preconditioned by small molecules to create amicable hepatic microenvironments, including endothelial disruption, microcirculatory dilation, instant blood-mediated inflammatory reaction (IBMIR) inhibition, and cell proliferation, which favor the engraftment and repopulation of the transplanted hepatocytes. Created in BioRender. Yind, K. (2024) BioRender.com/e30g211.

## 2 Signaling pathways and transcription factors in liver development

The manipulation of hepatocyte fate by small molecules relies on the understanding and recapitulation of liver development. In this section, we will elaborate on the role of signaling pathways and transcription factors in liver development. Under the activation of TGFβ signaling by Nodal/Activin A and canonical Wnt/β-catenin signaling, the primitive steak specifies into three germ layers, endoderm, mesoderm, and ectoderm (Camacho-Aguilar et al., 2024; Hayes et al., 2021). Combination of low concentrations of bone morphogenic protein (BMP), high concentrations of Nodal/Activin A, and Wnt/β-catenin signaling generates definitive endoderm (DE) (Pour et al., 2022). Wnt/β-catenin signaling promotes DE specification by induction of *NODAL* gene expression (Wild et al., 2020). As embryonic development proceeds, DE patterns into foregut, midgut, and hindgut. The liver and pancreas arise from posterior foregut (PFG) (Lotto et al., 2023). At mouse embryonic day (E) 8.0, a proportion of cells in ventral foregut proximal to mesodermal cells adopt a hepatoblast (HB) fate, being bipotent for hepatocytes and cholangiocytes (Lotto et al., 2023). HB specification is tuned by fibroblast growth factors (FGFs) and BMPs, secreted by cardiac mesoderm and septum transversum, respectively, while pancreatic fate is activated by TGFβ signaling (Lee et al., 2021). The hepatic signaling pathways inhibit pancreatic fate commitment and *vice versa* (Lotto et al., 2023). Additionally, retinoic acid and Wnt/β-catenin inhibition drive liver bud formation (Perugorria et al., 2019; Lotto et al., 2023). Proliferating HBs expand the liver bud based on cues from HGF, FGF, Wnt signaling, and Hippo signaling (Macchi and Sadler, 2020). Near E13.5, HBs commit to hepatocytes or cholangiocytes depending on periportal Notch and TGFβ signalings, which favor cholangiocytes differentiation whereas prevent hepatocyte differentiation (Yang et al., 2019). On contrary, Hippo signaling drive hepatocyte differentiation. Inhibition of Hippo signaling promotes cholangiocytes by increasing TGFβ signaling (Russell and Camargo, 2022). Together, temporally and spatially regulated signaling action patterns the hepatic lineage in a multi-step developmental process.

The concerted network of signaling pathways induces downstream transcription factors, including FOXA1-3, GATA4/6, TBX3, HEX, PROX1, HNF4α, HNF1α/β and C/EBPα/β, promoting hepatic differentiation (Tachmatzidi et al., 2021). The role of these transcription factors is evidenced by several studies which directly converted fibroblasts into hepatocytes by transducing specific combination of these transcription factors in fibroblasts (Garcia-Llorens et al., 2023; Rombaut et al., 2021). FoxA and Gata family are the ‘pioneer factors’ during liver bud formation. Prior to liver bud formation, FOXA1/2 and GATA4/6 bind to liver-specific genes and prime the compacted chromatin allowing other proteins to bind to their target genes (Tachmatzidi et al., 2021; Macchi and Sadler, 2020; Lee et al., 2005). Specifically, FOXA proteins bind to nucleosome and upregulate the levels of histone three lysine 4 (H3K4) me1 by recruiting lysine-specific demethylase 1 (LSD1) (Tachmatzidi et al., 2021). During HBs specification, the pioneer factors recruit EZH2, a histone methyltransferase (KMTs) that catalyzes histone three lysine 27 (H3K27) me3, to silent pancreatic-specific genes in HBs (Dumasia and Pethe, 2020). Different from FOXA1/2, FOXA3 expression delays to E10.5 and remains high level throughout liver development (Tachmatzidi et al., 2021), which exerts unique functions in liver-specific gene activation by interacting and travelling with RNA polymerase II from the distal to proximal regions of transcription start sites (Horisawa et al., 2020). HNF1β and HNF4α also play a crucial role in hepatic differentiation. HNF1β cooperates with GATA6 to upregulate HNF4α expression (Lau et al., 2018), which recruits RNA polymerase II to hepatic genes during liver bud formation (DeLaForest et al., 2018). By adulthood, HNF1β expression is substituted by HNF1α interacting with HNF4α (Tachmatzidi et al., 2021), which maintains hepatocyte identity and metabolic functions together with FOXA, TBX3, and CEBPα (Kotulkar et al., 2023).

Together, the liver development requires complicated networks of signaling pathways and transcription factors. The special and temporal regulation of Wnt/β-catenin, TGFβ, FGF, BMP, and HGF as well as the liver-specific transcription factors including FOXA and HNFs are crucial in liver development. Hepatic differentiation and reprogramming needs initiation and reactivation of these mediators, respectively.

## 3 Small molecule induction yields cell sources for HTx

A main hurdle of HTx is the limited availability of high-quality cells for transplantation. Attention has turned to using stem cell-derived hepatocytes since pluripotent stem cells (PSCs) and mesenchymal stem cells sources are abundant. Methods using combinations of signaling modulators have been developed to derive specific lineages by mimicking embryonic development. This review focuses on signaling pathways and epigenetic changes involved in hepatic differentiation and reprogramming and their chemical modulations (Figure 1).

### 3.1 Signaling modulation for hepatic differentiation

By mimicking the development signaling, stepwise chemical treatment induces differentiation of human embryonic stem cells (hESCs), induced PSCs (iPSCs), and human PSCs into hepatocytes. The target and mechanism of small molecules are summarized in Table 1. GSK3β inhibitor CHIR99021 (CHIR) activated Wnt/β-catenin to induce DE from hESCs combined with Nodal signaling activator activin A in RPMI + B27 medium (Raggi et al., 2022). Another GSK3β inhibitor BIO was also used to activate Wnt/β-catenin pathways (Tasnim et al., 2015; Onai, 2019). Activin A is able to be substituted with IDE1, an activator of Nodal signaling (Luo et al., 2021). The combination of IDE1, CHIR, and LY294002 (a PI3K inhibitor) induced DE specification from human umbilical cord-derived mesenchymal stem cells (Luo et al., 2021). However, this combination induced mesoderm formation in human PSCs probably due to the excessive level of Wnt signaling favoring mesoderm formation (Pan et al., 2022). To cope with, PD0332991, a CDK4/6 inhibitor was added to the combination of IDE1 and CHIR, resulting in more specific DE differentiation (Pan et al., 2022). To promote DE differentiation to PFG, Touboul T et al. treated hESCs with Wnt/β-catenin inhibitor IWR-1 together with activin A and noggin, resulting in induction of PFG gene expression (Touboul et al., 2016), although noggin was later found to impede with the promoting effect of IWR-1 on PFG induction. For further differentiation into HBs, a combined protocol of BMP4, IWR-1, and TGFβ inhibitor SB431542 eliciting hepatic markers, including fetoprotein (AFP), albumin (ALB), and prospero homeobox 1 (PROX1) was established (Touboul et al., 2016). Du, C et al. combined A83-01, a TGFβ signaling inhibitor, with two epigenetic modulators sodium butyrate (NaB) and dimethyl sulfoxide (DMSO) to induce hepatic differentiation from DE (Du et al., 2018). Finally, Touboul T et al. showed that CHIR treatment generated proliferative HBs with co-expression of HNF6 and PROX1, although activation of Wnt/β-catenin pathways theoretically promote biliary differentiation (Touboul et al., 2016).

**TABLE 1:** Small molecules in hepatic differentiation and reprogramming.

Signaling pathway/Target	Small molecule	Mechanism	References	Clinical trial
GSK3β inhibitor	CHIR99021	Promote PSC differentiation into DE Convert MEF to hepatocyte HPC expansion and self maintenance Convert hepatocyte to HPC Maintain hepatocyte function	Raggi et al. (2022), Tasnim et al. (2015), Onai (2019) Guo et al. (2017) Lv et al. (2015), Pan et al. (2019) Wu et al. (2017), Jiang et al. (2023) Chen et al. (2021)	-
Tankyrase inhibitor	IWR-1	Promote DE differentiation into PFG Promote PFG differentiation into HPC	Touboul et al. (2016)	-
TGFβ inhibitor	SB431542	Promote PFG differentiation into HPC Maintain hepatocyte function	Touboul et al. (2016) Xiang et al. (2019), Chen et al. (2021)	-
	A83-01	Promote DE differentiation into HPC Promote HPC differentiation into hepatocyte HPC expansion and self maintenance Convert hepatocyte to HPC	Du et al. (2018) Du et al. (2018) Lv et al. (2015), Pan et al. (2019) Wu et al. (2017)	-
	RepSox/E−616452	Convert MEF to hepatocyte HPC expansion and self maintenance	Guo et al. (2017), Li et al. (2017a) Lv et al. (2015)	-
PI3K inhibitor	LY294002	Promote MSC differentiation into DE Maintain hepatocyte function	Luo et al. (2021) Sun et al. (2019)	-
ROCK inhibitor	Y-27632	Convert hepatocyte to HPC Maintain hepatocyte function	Wu et al. (2017), Jiang et al. (2023) Chen et al. (2021)	NCT05309135 NCT06041256
cAMP signaling agonist	Forskolin	Convert MEF to hepatocyte HPC expansion and self maintenance Maintain hepatocyte function	Li et al. (2017b) Pan et al. (2021) Lin et al. (2023)	NCT01254006
HDAC1 inhibitor	Sodium butyrate	Promote DE differentiation into HPC Convert MEF to hepatocyte	Tasnim et al. (2015), Du et al. (2018) Zhu et al. (2014)	NCT04415333 NCT05808985 NCT05456763
HDAC inhibitor	Valproic acid	Convert MEF to hepatocyte	Li et al. (2017a)	NCT06248931 NCT04698525 NCT03885154
Unknown probably mediate histone acetylation	Dimethyl sulfoxide	Promote DE differentiation into HPC Convert MEF to hepatocyte	Du et al. (2018) Zhu et al. (2014)	FDA approved for the symptomatic relief of interstitial cystitis NCT05223244
LSD1 inhibitor	Parnate	Convert MEF to hepatocyte HPC expansion and self maintenance Maintain hepatocyte function	Guo et al. (2017), Li et al. (2017b), Zhu et al. (2014)	FDA approved for major depressive episode without melancholia NCT01430455 NCT02273102

Abbreviation: PSC, pluripotent stem cell; DE, definite endoderm; MEF, mouse embryonic fibroblast; HPC, hepatic progenitor cell; MSC, mesenchymal stem cell.

Hepatocyte growth factor (HGF) and its receptor c-Met regulates hepatic maturation during liver development. In developing livers, the expression of HGF and c-Met upregulated between 4 and 21 days after birth (Hu et al., 1993). In fetal hepatocytes, treatment of HGF together with glucocorticoids promoted the expression of ALB (Kamiya et al., 2001), suggesting the effect of HGF on hepatic maturation. N-hexanoic-Tyr-Ile-(6) aminohexanoic amide (Dihexa), a small-molecule angiotensin IV analog, was found to efficiently activate HGF/c-Met pathway (Benoist et al., 2014). Treatment of Dihexa and glucocorticoids successfully induced functional HLCs from hepatic progenitor cells (HPCs)(Siller et al., 2015; Asumda et al., 2018). In addition, HGF can be replaced by other small molecules. Shan, J et al. identified two chemical compounds functional hits 1 (FH1) and functional proliferation hit 1 (FPH1) through high-throughput screen (Shan et al., 2013). Later, Du, C et al. established a protocol including FH1, FPH1, A83-01, and glucocorticoids, which yielded functional HLCs more efficiently compared to growth factor groups (Du et al., 2018). Luo S et al. combined FH1 with growth factors to induce hepatocyte differentiation from HPCs. FH1 induced hepatocytes with higher efficiency and similar maturity compared to HGF induced ones (Luo et al., 2021).

Recently, the hepatic differentiation of current good manufacturing practice (cGMP) compliant human iPSC (hiPSC) and hESC lines have been validated. The protocol successfully generated hepatocytes from cGMP cell lines using small molecules combined with growth factors in 21 days (Blackford et al., 2019). The *ALB* expression was detected but insignificant in the induced hepatocytes, suggesting that the hepatocytes was immature (Blackford et al., 2019). In comparison, the pure chemical protocol from Du et al. produced hepatocytes from hESCs within 13 days. The *ALB* positive hepatocytes account for 67.7% *versus* 37.1% in the growth factor group (Du et al., 2018). The hepatic differentiation using growth factor-free small molecule cocktails should also be validated in cGMP compliant hiPSC and hESC lines for future clinical application. Moreover, the combination and dosage of small molecules vary between research groups. Comparison studies are needed to evaluate the differentiation efficiency of protocols and to conclude a cGMP compliant protocol.

### 3.2 Small molecules for direct reprogramming to hepatic lineage

Recently, studies have shown that somatic cells can be directly reprogrammed into specific cell lineage, including hepatocytes. Direct reprogramming without entering pluripotent cell state shortens the time for cell differentiation and lower the risks of tumorigenesis, which is deemed as a promising method to yield hepatocytes for HTx.

Early in 2011, Sekiya et al. established three combinations comprising two transcription factors: HNF4α with FOXA1, FOXA2 or FOXA3, which successfully converted mouse embryonic fibroblasts (MEFs) into induced hepatocyte-like cells (iHeps) (Sekiya and Suzuki, 2011). However, the conversion efficiency of the protocol was insufficient (only 0.3%) (Sekiya and Suzuki, 2011). Hui’s group discovered a specific combination of three transcription factors consisting of GATA4, HNF1α and FOXA3, which converted p19^Arf−/−^ mouse tail-tip fibroblasts to iHeps with a higher conversion efficiency (Huang et al., 2011; Ji et al., 2013). The researchers suggested that *Hnf1a* and *Foxa3* transduction was already sufficient to induce iHeps and addition of *Gata4* enhanced the conversion efficiency (Ji et al., 2013). Later, Hui et al. showed that transduction of *Hnf1β* and *Foxa3* directly induced MEFs into hepatic stem cells which had the bipotential of differentiating into hepatocytes and cholangiocytes (Yu et al., 2013).

The clinical application of iHep generated by viral transduction in HTx have been limited due to the safe uncertainty, genetic instability and tumorigenesis risk. Therefore, studies about chemical induction of hepatocytes from fibroblasts have thrived in the field (Tables 1, 2).

**TABLE 2:** Small molecules in promoting hepatocyte engraftment.

Pharmacological mechanism/Target	Small molecule	Mechanism of promoting cell engraftment	References	Indication/Clinical trial
Intercalation into DNA Topoisomerase II Inhibition Generation of Free Radicals Apoptosis Induction	Doxorubicin	Induce hepatic endothelial injury	Kim et al. (2005)	FDA approved for various neoplasms (e.g., breast and ovarian cancer)
Inhibition of proinflammatory cytokine release Apoptosis Induction Angiogenesis inhibition	Thalidomide	Induce hepatic endothelial injury Inhibit kupffer cell and proinflammatory cytokine release	Viswanathan et al. (2016)	FDA approved for multiple myeloma and erythema nodosum leprosum
Guanylate cyclase activator	Nitroglycerine	Dilate hepatic sinusoid Inhibit kupffer cell Ameliorate endothelial injury	Slehria et al. (2002) Bahde et al. (2013)	FDA approved for angina pectoris, peri-operative hypertension , and congestive heart failure
Unselective endothelin receptor antagonist	Bosentan	Increase VEGF release and prevent TNF-α- or H2O2-induced cytotoxicity	Bahde et al. (2013)	FDA approved for pulmonary arterial hypertension
Endothelin receptor A antagonist	Darusentan	Dilate hepatic sinusoid Inhibit kupffer cell Ameliorate endothelial injury	Bahde et al. (2014)	NCT00330369 NCT00389779
Antioxidant Mucolytic Activity Reduce coagulation factor activity	N-acetyl-L-cysteine	Impair procoagulant activity of hepatocyte Reduce cell apoptosis	Stéphenne et al. (2007) Heil et al. (2018)	FDA approved for mucolytic therapy, antidote for acetaminophen overdose , and acute or subacute hepatic failure
Prothrombin inhibitor	Bivalirudin	Impair procoagulant activity of hepatocyte	Stephenne et al. (2012)	FDA approved for unstable angina after percutaneous transluminal coronary angioplasty
Prothrombin inhibitor	Dabigatran	Upregulate thrombomodulin levels Protect sinusoidal endothelial cells Inhibit kupffer cell and proinflammatory cytokine release	Noguchi et al. (2021)	FDA approved for venous thromboembolism and atrial fibrillation
C5a receptor 1 antagonist	PMX53	Inhibit kupffer cell and proinflammatory cytokine release Inhibit platelet aggregation	Kusakabe et al. (2020)	-
ROCK inhibitor	Ripasudil	Block membrane attack formation and inhibit Kupffer cell	Ma et al. (2023)	NCT04620135

Guo, R et al. showed that combination of a single transcription factor FOXA1, FOXA2, or FOXA3 with six compound cocktails, including CHIR, RepSox, valproic acid (VPA), Parnate, TTNPB, Dznep (termed CRVPTD), directly induced MEFs into iHeps (Guo et al., 2017). Researchers postulated that FOXAs upregulated liver-specific genes independent of any specific signaling pathway and thus was difficult to replace. Horisawa, K et al. demonstrated that liver-specific gene expression was promoted in MEFs by sequential and cooperative binding of FOXAs and HNF-4α to chromatin. FOXA3 exert unique regulations on the gene expressions among FOXA proteins, that is, by binding to and co-transversing the target genes with RNA polymerase II, which is indispensable for reprogramming MEFs to hepatocytes (Horisawa et al., 2020).

Later studies developed protocols independent of ectopic expression for direct reprogramming. Li, X et al. established a seven-compound cocktail (VPA [V], TD114-2 [T], E−616452 [6], tranylcypromine/Parnate [P], forskolin [F], AM580 [A], and EPZ004777 [E]) to regulate reprogramming-related signaling pathways (T6FA) and to modulate the epigenetic profile (VPE) (Li et al., 2017a). The small-molecule cocktail induced extra-embryonic endoderm (XEN)-like cells from fibroblasts bypassing the pluripotent state, which could differentiate towards hepatocytes (Li et al., 2017b). A later study elucidated that compounds CHIR, E−616452, and forskolin firstly worked in a cooperative manner to activate *Sox7*; CHIR/forskolin and E−616452 then activated *Gata4* and *Sall4* expression, respectively. The consecutive activation of the crucial transcription factors attributed to the conversion of MEF to XEN-like cells (Yang et al., 2020). Bai, Y et al. established a two-stage chemical cocktail to directly reprogram MEFs to iHeps (Bai et al., 2023), modified from the cocktails (C6FAE and C6F5UE) presented in Yang’s work (Yang et al., 2020). The researchers found that addition of vitamin C in the second stage was the optimal protocol to induce iHeps, with the iHep subpopulation of 15% in total cells (Bai et al., 2023). Principal component analysis and RNA-seq showed that the iHeps resembled PHHs (Bai et al., 2023). Finally, the iHeps was capable of engrafting the liver in Fah^−/−^ mice and improving liver functions *in vivo* in a similar manner to PHHs (Bai et al., 2023). Noteworthy, Zhong Z et al. directly reprogramed MEFs into iHeps with one step chemical induction consisting of SB431542, CHIR99021, BIX01294 (G9a KMT inhibitor), LDN193189, and DAPT (Zhong et al., 2024). During 12-day induction, no pluripotency gene expression (*Oct4, Sox2, and Nanog*), immature HB markers, and HPC markers were detected, suggesting that the conversion of MEFs to iHeps bypassed the intermediate stem cell stage (Zhong et al., 2024). Mechanistically, the chemical cocktail suppressed SNAI1 expression, which induced mesenchymal to epithelial transition and HNF4α expression, thereby promoting iHep generation (Zhong et al., 2024).

Hui’s group reported that the induction of iHep generated by transcription factor cost 14 days and the induction efficiency was around 23% (Huang et al., 2011). In comparison, in studies using chemical approaches to produce iHep, the induction time was 20, 12, 12 days and the efficiency was over 20%, 15%, and 80%, respectively (Li et al., 2017a; Bai et al., 2023; Zhong et al., 2024). Collectively, the chemical approach inducing iHep from fibroblasts has a similar and even more robust induction efficiency to genetic approach, making it a promising method to produce hepatocytes from MEFs.

### 3.3 Epigenetic modulation for differentiation and reprogramming

Epigenetic modulations are important mechanisms of transcriptional regulation during normal development. During differentiation from PFG towards HBs, histone acetylation significantly increased at the liver regulatory elements mediated by histone acetyltransferase P300 (Xu et al., 2011). Moreover, H3K27 KMTs enriched at *Pdx1* gene (a pancreatic gene) upstream which suppressed the pancreatic development while favored hepatic development (Xu et al., 2011). The methylation of CpG islands is regulated by DNA methyltransferases (DNMTs) and mediate gene expressions by mediating promoters (Wilkinson et al., 2023). Based on the mechanisms, histone deacetylase (HDAC) inhibitors and DNA/histone methylation mediators are used to promote hepatic differentiation. It was reported that combination of NaB with DMSO elicited hepatic differentiation from DE with comparable hepatic marker expression (AFP and HNF4α) to the growth factor-treated group (Tasnim et al., 2015). Demethylation of CpG by DNMT inhibitors allowed liver-specific gene expression, including AFP and ALB (Snykers et al., 2009). It was reported that combination of nanaomycin A, a selective DNMT3B inhibitor, with FGF4 and BMP4 promoted the differentiation from DE to HPCs (Nakamae et al., 2018).

Epigenetic modulation is also required in cell reprogramming. In differentiated cells, high levels of DNA methylation occurred in the CpG-rich promoters of pluripotency-related genes (Baral et al., 2022; Ghazimoradi and Farivar, 2020). Therefore, inhibition of DNMTs promotes demethylation of CpG islands and thereby removes the epigenetic barrier of cell reprogramming. It was demonstrated that the combination of RG108 (a DNMT inhibitor) and with BIX01294 promoted the reprogramming of MEFs to iPSC upon Oct4 and Klf4 transduction (Shi et al., 2008).

Histone acetylation/deacetylation is mediated by histone acetyltransferases and HDACs, respectively. Histone acetylation is related to open chromatin structure and transcriptional activation (Baral et al., 2022). HDAC inhibitors, including VPA and NaB, were shown to facilitate cell reprogramming in several studies (Aguirre-Vázquez et al., 2021; Duan et al., 2019; Mali et al., 2010). Histone lysine methylation is tuned by KMTs and histone lysine demethylases. The effect of histone lysine methylation on gene transcription varies, either activating or inhibitory. LSD1 specifically catalyzes the demethylation of H3K4/9me1/2 (Dai et al., 2021). Enhancer of zeste homolog 2 (EZH2) and DOT1L catalyzes methylation of H3K27 and histone three lysine 79 (H3K79), respectively (Wu et al., 2023; Lee et al., 2022). Small molecule inhibitor of LSD1 (Parnate), EZH2 (DZNep), and DOT1L (EPZ004777) promoted somatic reprogramming efficiency (Martinez-Gamero et al., 2021; Liuyang et al., 2023).

Based on the aforementioned mechanisms, researchers have applied small molecules to modulate epigenetic profiles in somatic cells and thus to promote somatic reprogramming into hepatocytes bypassing the iPSC stage (Table 1 and 2). Zhu, S et al. demonstrated that combination of NaB, Parnate, and RG108 with a Wnt signaling activator significantly reprogrammed MEFs into multipotent progenitor cells with transient expression of three transcription factors (OCT4, SOX2 and KLF4) (Zhu et al., 2014). Multipotent progenitor cells differentiated towards EPCs and hepatocytes sequentially in the hepatocyte induction medium (Zhu et al., 2014). Guo R et al. used VPA, Parnate, TTNPB, and Dznep combining with other small molecules to induce direct reprogramming from MEFs to iHeps with only a single factor FOXA3 (Guo et al., 2017). Li, X et al. developed a 7-compound protocol consisting of VPA, EPZ004777, Parnate and other small molecules which directly induced the XEN-like cells from MEFs (Li et al., 2017b). Zhong Z et al. combined BIX01294 with other signaling modulators to directly converted MEFs to iHep (Zhong et al., 2024).

### 3.4 Small molecule modulation of cell expansion and maturation

HPCs can be yielded either from livers or iPSCs and thus becomes one of accessible cell sources for HTx. However, the limited ability of *ex vivo* proliferation impedes with its application. During liver development, Wnt signaling promotes the proliferation, expansion, and differentiation of HPCs (Perugorria et al., 2019). Hedgehog signaling and BMP4 facilitates the proliferation of fetal HPCs *in vitro* (Hirose et al., 2009; Wang et al., 2015), while TGFβ signaling remarkably inhibits HPC colony formation in vitiro (Clark et al., 2005). Notch signaling activation promotes HPC differentiation towards cholangiocytes (Duan et al., 2018). These data suggested that fine modulation of these critical signalings might contribute to HPC expansion.

Our lab established a protocol consisting of EGF, CHIR, E−616452 and two bioactive lipids lysophosphatidic acid and sphingosine 1-phosphate, hereafter termed as ECELS. Mouse HBs maintained self-renewal in the Matrigel-coated media containing ECELS and bovine serum albumin (Lv et al., 2015). These expandable HBs could differentiated to mature hepatocytes by chemical induction (Lv et al., 2015). Pan, T et al. established a chemical cocktail of CHIR, A8301, and SAG (a Hedgehog signaling activator) combined with EGF, HGF and BMP4, which significantly promoted the expansion and stemness maintenance of HPCs (Pan et al., 2019). Later, researchers replaced EGF, HGF, and BMP4 with forskolin, Dihexa, and vitamin C, respectively. This chemical cocktail enabled the long-term self-renewal with efficient proliferation rate for at least 20 passages and the capability of differentiating into hepatocyte and cholangiocytes (Pan et al., 2021).

Mature hepatocytes converted to liver progenitor-like cells and re-differentiated into hepatocytes in response to chronic periportal liver injuries *in vivo* (Li et al., 2020). The liver progenitor-like cells generated duct-like cells when liver damage persisted (Li et al., 2020). Yan et al. established a transition and expansion medium which consisted of EGF, HGF, CHIR, lipids lysophosphatidic acid, sphingosine 1-phosphate, A83-01, and Y-27632. This protocol elicited significant transition of hepatocytes to duct-like cells and promoted the proliferation of duct-like cells (Wu et al., 2017). Miyoshi T et al. converted PHHs harvested from cirrhotic livers to liver progenitor cells by a protocol including Y-27632, A83-01, and CHIR (YAC). The induced liver progenitor cells differentiated into mature hepatocyte under induction of YAC, oncostatin M, and dexamethasone (Miyoshi et al., 2022).

The quick loss of functions in isolated hepatocytes is one of the main hurdles in hepatocyte-based cell therapy. Therefore, it is urgent to investigate on the underlying mechanism and discover corresponding strategies. Our lab demonstrated that mechanical tension represented by actin remodulation elicited hepatocyte dedifferentiation towards HPCs through Yap activation. Yap deletion in isolated hepatocytes resulted in maintenance of ALB and HNF4α expression (Sun et al., 2019). Based on the iterative chemical screening, we found that combination of an actin polymerization inhibitor Latrunculin B and actomyosin contraction inhibitor Blebbistatin led to increased levels of ALB and HNF4α. Addition of Dasatinib, XAV939, and LY294002 (Yap mediators) further facilitated hepatic genes expression (Sun et al., 2019). This chemical cocktail (termed LBDXL) was able to maintain the hepatic gene expression and metabolic functions of hepatocytes cultured in Matrigel-coated medium for up to 3 weeks (Sun et al., 2019).

The activation of epithelial to mesenchymal transition in hepatocytes results in loss of normal hepatic functions and regenerative capacity, where TGF-β signaling pathway play an important role (Xue et al., 2013). Xiang C et al. confirmed that the expression of components of TGF-β signaling pathway significantly upregulated in cultured PHHs. The researchers established a 5-compound (5C) protocol, consisting of SB431542, forskolin, DAPT (Notch inhibitor), IWP2 (Wnt inhibitor), and LDN193189 (BMP inhibitor) to suppress the expression of epithelial to mesenchymal transition marker genes (Xiang et al., 2019). 5C-cultured PHHs still supported hepatitis B virus infection after 4-week culture (Xiang et al., 2019). Additionally, Chen Y et al. developed a small-molecule cocktail consisting of SB431542, acetylcysteine (ROS inhibitor), CHIR, and Y-27632 (SACY)(Chen et al., 2021). Compared to 5C culture system, SACY exhibited higher level of ALB and α1 antitrypsin production and urea synthesis (Chen et al., 2021).

Loss of functions in hepatocytes was also observed *in vivo*, specifically in chronically injured liver. Lin P et al. showed that damaged hepatocytes harvested from CCl4-induced mice liver regained hepatocyte phenotypes and functions with treatment of five compounds (forskolin, CID755673, GSK429286A, ETC-1002, and phenylpropanoid glycoside) (Lin et al., 2023). Injection of revitalized hepatocytes promoted liver regeneration in CCl4-induced liver injury (Lin et al., 2023).

## 4 Small molecules improve hepatocyte engraftment and repopulation

**FIGURE 2:**
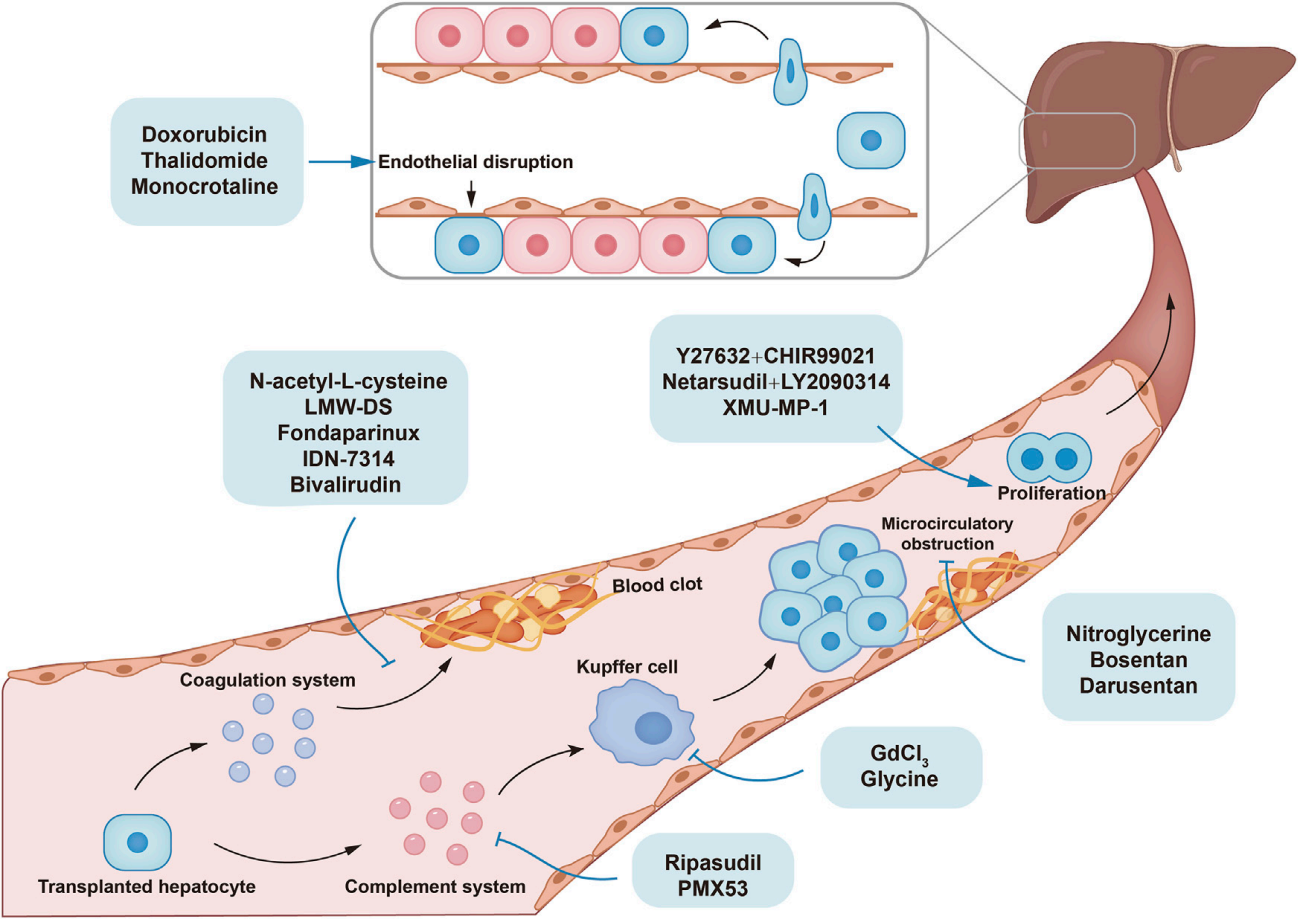
The mechanisms of small molecules improving poor cell engraftment and repopulation in hepatocyte transplantation.

Low cell engraftment and repopulation rate is another hurdle of HTx. To improve hepatocyte engraftment, preconditioning strategies including irradiation and partial hepatectomy have been established but chemical intervention provide a less invasive approach, which are reviewed in the following section. The potential mechanisms and clinical trials of small molecules improving poor cell engraftment and repopulation in hepatocyte transplantation were shown in Figure 2; Table 2.

The transplanted hepatocytes are cleared by instant blood-mediated inflammatory reaction (IBMIR) in the early period after HTx. Coagulation system, complement system, and innate immune cells are important components involved in IBMIR. Microcirculatory obstruction also participates in IBMIR by activating Kupffer cells. Advantageous proliferation of transplanted cells improves liver repopulation. N-acetyl-L-cysteine (NAC), low-molecular-weight dextran sulfate (LMW-DS), fondaparinux, and bivalirudin inhibit the coagulation system, while PMX53, ripasudil, gadolinium chloride (GdCl_3_), and glycine inhibit innate immune system, thereby alleviating hepatocyte clearance. Nitroglycerine, bosentan, and darusentan mitigate microcirculatory obstruction by dilating hepatic sinusoid. XMU-MP-1 enhanced cell proliferation by targeting Hippo pathway. Combination of Y27632 and CHIR99021, or netarsudil and LY2090314 improved liver repopulation *in vivo* by promoting the dediffereantiation and redifferentiation of hepatocytes.

### 4.1 Disruption of endothelial integrity in liver

Gupta et al. observed disrupted sinusoidal endothelium structure at 24 h post-HTx, suggesting endothelial disruption permits transplanted cell entrance into liver parenchyma (Gupta et al., 1999b; Gupta et al., 1999a). Therefore, facilitating sinusoidal endothelium disruption can enhance engraftment efficiency by promoting the entrance of hepatocytes into liver plates (Figure 2).

Small-molecule drugs including monocrotaline, doxorubicin, rifampicin, and phenytoin increased cell engraftment *versus* controls by causing hepatic endothelial damage (Joseph et al., 2006; Kim et al., 2005; Wu et al., 2008). Thalidomide has the anti-inflammatory properties via selective inhibitory activity of tumour necrosis factor-α (TNFα) and the anti-angiogenesis effect via downregulating VEGF levels. (Amare et al., 2021; Barbarossa et al., 2022). Viswanathan et al. found that Thalidomide was able to induce transient upregulation of serum hyaluronic acid levels *versus* control, suggesting the rapid endothelium damage (Viswanathan et al., 2016). Thalidomide pretreatment markedly upregulated transplanted cell numbers in liver *versus* non-treatment groups (Viswanathan et al., 2016), also improving engraftment via immune regulation discussed later. It is speculated that the effect of Thalidomide on hepatic endothelial damage is related to its anti-angiogenesis property. Thalidomide inhibited proliferation of human umbilical vein endothelial cells *in vitro* probably via downregulation of transcription factor SP1 (Moreira et al., 1999). Another study demonstrated that Thalidomide suppressed VEGF production by human peritoneal mesothelial cells and thereby inhibited endothelial tube formation via STAT3/SP4 signaling (Zhu et al., 2021). These data offer a mechanistic insight of how Thalidomide disrupt hepatic endothelial integrity, although further studies are needed.

In conclusion, using small molecules to induce endothelial disruption benefits cell engraftment. Mechanistically, endothelial disruption increases sinusoid permeability, allowing hepatocytes to pass the endothelial barrier and enter liver parenchyma.

### 4.2 Prevention of hepatic microcirculatory obstruction

Slehria et al. observed sinusoidal blood flow cessation in liver after HTx, persisting over 3 h and ameliorating by 24 h (Slehria et al., 2002). Researchers suggested that the overall sinusoidal perfusion declined from 94% to 84% on day 1% and to 76% on day 7 after HTx (Wilhelm et al., 2004), suggesting transplanted hepatocytes cause immediate and long-term disruption to sinusoidal blood flow and microcirculation.

Hepatic microcirculatory obstruction affects transplanted cell distribution. Cells entering sinusoids and integrating into liver plates avoid immune clearance, while those remaining in portal areas confront clearance (Gupta et al., 1999b; Koenig et al., 2005). Rajvanshi P et al. suggested hepatocyte location relates to portal vein radicles size. The intrasplenically-transplanted hepatocytes were largely located in periportal area (zone 1) in mice, while in downstream midlobular (zone 2) or perivenous (zone 3) areas in larger animals (Rajvanshi et al., 1996). Indeed, occlusion is more likely in smaller vessels *versus* larger ones, hindering sinusoidal distribution while increasing portal areas exposure, leading to immune clearance of transplanted hepatocytes.

Hepatic microcirculatory obstruction causes ischemia-related cell injury in the liver. After HTx, transient ischemia in portal areas leads to an immediate sinusoidal blood flow cessation (Wilhelm et al., 2004; Slehria et al., 2002). Wilhelm A et al. observed markedly increased Kupffer cells (KCs) on day 1 post-HTx (Wilhelm et al., 2004), which release ROS and proinflammatory cytokines including TNF-α, IL-1, IFN-γ (Peralta et al., 2013). ROS induces transplanted cell injury by inducing mitochondria dysfunction, protein synthesis disruption and cell membrane damage (Bardallo et al., 2022). The proinflammatory cytokines promote neutrophil and peripheral macrophage infiltration, which further enhance the immune reaction. Indeed, Gupta S et al. observed the infiltration of OX-43 antibody-positive granulocytes, phagocytes, and activated macrophages in portal areas (Gupta et al., 1999b). Overall, ischemia activates the immune response, leading to transplanted cell injury.

This evidence suggests that hepatic microcirculatory obstruction interferes with cell distribution and triggers immune responses. Thus, ameliorating the hepatic microcirculation with vascular dilators is a promising method to improve transplanted hepatocyte engraftment.

Nitroglycerine relaxes vascular smooth muscle cells and cause vascular dilation by increasing NO levels and activating guanylate cyclase. Slehria S et al. first reported that nitroglycerine treatment in rats significantly reduced periportal blood flow perturbation after HTx and increased transplanted hepatocytes in the liver and specifically in zone 2 at 2h after HTx but not long-term (Slehria et al., 2002), showing sinusoid dilatation contributed to hepatocyte distribution but not translocation. Other mechanisms may play a role in hepatocyte entry into liver plates. In another study, nitroglycerine significantly decreased KC activation and endothelial injury (Bahde et al., 2013), both of which are crucial in preventing ischemia-reperfusion injury. Bosentan (BOS), an unselective endothelin receptor blocker, increased desmin + hepatic stellate cells (HSCs) numbers and VEGF release of HSCs in rat (Bahde et al., 2013), potentially aiding cell entry via increasing hepatic sinusoid permeability. Moreover, conditioned medium of BOS-treated HSCs containing BOS and VEGF prevented TNF-α- or H_2_O_2_-induced cytotoxicity against hepatocytes (Bahde et al., 2013). BOS-incubated cells engrafted 2-fold higher than controls while systemic treatment of BOS in recipient rats did not (Bahde et al., 2013). A selective endothelin-1 receptor A blocker, Darusentan (DAR), was effective in dilating hepatic sinusoids, which allowed greater entry of hepatocytes and thus enhanced cell engraftment. Moreover, DAR reduced endothelial injury, KC activation and hepatic ischemia (Bahde et al., 2014). Contrary to BOS, DAR failed to prevent cytotoxicity and negatively affect hepatocyte proliferation *in vitro* (Bahde et al., 2014), suggesting that DAR favors systemic use. Noteworthy, the different effect of BOS and DAR on hepatocytes and host indicates the endothelin receptor A or B signaling pathway exert distinct biological functions in the liver, whereas the underlying mechanism warrants further investigations.

### 4.3 Inhibition of instant blood-mediated inflammatory reaction (IBMIR)

IBMIR was first described in islet transplantation which cause instant islet loss (van der Windt et al., 2007; Mou et al., 2022; Yan et al., 2022), involving interactions between platelet aggregation, coagulation system, complement system, and neutrophil/macrophage infiltration (Kale and Rogers, 2023). Hepatocyte-induced IBMIR occurs both *in vitro* and patients receiving HTx and is a dominant factor contributing to early cell loss of HTx (Lee et al., 2016).

Tissue factor (TF) is the initiating factor of coagulation extrinsic pathway, which expressed on cell membrane of mice and human hepatocytes (Kopec and Luyendyk, 2014). In the tubing loop system model, infusion of 5 × 10^5^ hepatocytes into the whole-blood significantly increased D-dimer levels and decreased platelet counts (Stéphenne et al., 2007). The increase of D-dimer levels was also observed in a Crigler-Najjar patient after the first infusion of hepatocytes (Stéphenne et al., 2007). The changes were prevented by anti-TF monoclonal antibody, suggesting the procoagulant activity of hepatocytes depended on TF (Stéphenne et al., 2007).

Complement system can be directly activated by hepatocytes through antibody recognition, lack of membrane-bound regulators, and extracellular matrix proteins exposure (Nilsson et al., 2014), and indirectly by the crosstalk with coagulation system. For example, fXa and thrombin can convert C3 into C3a (Dzik, 2019). TF-induced thrombin cleaves C5 at a novel R947 site and forms new fragments, C5_T_ and C5b_T_ (Krisinger et al., 2012). Reciprocally, complement proteins interact with coagulation system. C5a can elicit significant increase in TF expression of endothelial cells (Dzik, 2019). Activation of coagulation and complement contributes to the infiltration of neutrophils, monocytes and macrophages, resulting in hepatocyte loss.

Collectively, TF-initiated coagulation activates the IBMIR with interactions between coagulation, complement system, and immune response promote IBMIR process. Interventions with each step with small molecules can attenuate IBMIR and enhance cell engraftment (Figure 2).

#### 4.3.1 Inhibition of coagulation system

N-acetyl-L-cysteine (NAC), used in treating paracetamol overdose as an antioxidant, decreased coagulation factor activity and delayed prothrombin time (PT) *in vitro* (Knudsen et al., 2005; Jang et al., 2013; Pizon et al., 2011). Although the mechanism of anti-coagulation property of NAC is elusive, some studies offer a possible mechanistic insight. In a study, NAC markedly reversed the procoagulant activity of monocyte-derived microvesicles induced by high glucose levels via inhibiting p38/MAPK signaling (Li et al., 2017a). In HTx, Stéphenne X et al. reported that NAC impaired the TF-dependent procoagulant activity of hepatocytes in a dose-dependent manner, preventing platelet depletion and D-dimer increase (Stéphenne et al., 2007). Moreover, under hypoxia environment inevitable in HTx due to cell occlusion, NAC treatment increased the Bcl-2/Bax ratio and vital hepatocytes (Heil et al., 2018). Reactive oxygen species (ROS) are produced during hypoxia environment, NAC, as an ROS inhibitor, mitigated oxidative stress and downregulated apoptotic factors during hepatic ischemia-reperfusion injury (IRI) (Singh et al., 2024). With its dual functions promoting engraftment by interfering coagulation and enhancing viability, NAC shows promise as a small molecule for clinical HTx application.

Low-molecular-weight dextran sulfate (LMW-DS), a type of glycosaminoglycans, has been reported to inhibit coagulation and complement system. LMW-DS inhibited fXIa and C1s by potentiating C1 inhibitor (Wuillemin et al., 1997; Mauron et al., 1998; Wuillemin et al., 1996) and prevented the deposition of C3 and C4 (Wuillemin et al., 1997). LMW-DS impaired fXa activity and prolonged PT, activated partial thromboplastin time, and thrombin time in human plasma (Drozd et al., 2017). Based on these, LMW-DS has been used in inhibition of IBMIR in animal models and patients of islet transplantation (von Zur-Mühlen et al., 2019; Naziruddin et al., 2014; Kourtzelis et al., 2015), showing similar efficacy and safety with heparin (von Zur-Mühlen et al., 2019). Noteworthy, Gustafson EK et al. reported that LMW-DS remarkably prevented hepatocyte-induced IBMIR, with a maintenance of platelet count, decrease of thrombin-antithrombin complex (TAT), and inhibition of complement system (Gustafson et al., 2011; Gustafson et al., 2017). Mechanistically, LMW-DS inhibited the fibrin-induced activation of contact activation pathway and the lectin complement pathway (Gustafson et al., 2017). Compared to heparin, LMW-DS more effectively inhibited coagulation and complement cascades (Gustafson et al., 2017). Therefore, LMW-DS is a promising agent against hepatocyte-induced IBMIR.

Small molecule inhibitors of coagulative factors have also been investigated to prevent IBMIR in cell transplantation. Fondaparinux (antithrombin activator) and the direct thrombin inhibitor drugs including hirudin and bivalirudin significantly inhibited procoagulant activity of hepatocytes *in vitro* (Stephenne et al., 2012). The effect of thrombin inhibitor on thrombo-inflammation has also been studied in hepatic IRI. Dabigatran treatment upregulated the thrombomodulin levels and reduced damage-associated molecular high-mobility group box-1 release from injured cells triggering inflammation (Noguchi et al., 2021). Through this mechanism, sinusoidal endothelial cells are protected from hypoxia-reoxygenation damage, indirectly preventing hepatocyte injury via paracrine effects of sinusoidal endothelial cells (Noguchi et al., 2021). In addition, dabigatran reduced the neutrophil infiltration and pro-inflammatory cytokines (Noguchi et al., 2021). As hypoxia-reoxygenation damage occurs in HTx due to cell occlusion, thrombin inhibition may hinder coagulation activation and inflammatory reaction triggered by hypoxia-reoxygenation damage in HTx.

Hepatocyte apoptosis is an important feature of acute and chronic liver diseases. Kopec AK et al. found that Fas-induced apoptosis increased the TF activity of mouse hepatocytes by upregulating caspase 3. Pretreating hepatocytes with IDN-7314, a pan-caspase inhibitor, impaired the TF activity and inhibit the coagulation activation *in vitro* and *in vivo* (Kopec et al., 2018). Although it is unclear whether the endogenous TF in liver microenvironment of recipients promotes IBMIR, a new sight into the relationship between coagulation and apoptosis is given for further studies.

#### 4.3.2 Inhibition of the innate immune system

The innate immune system, including complement system, cytokines, and innate immune cells, functions downstream of coagulation system and determines transplanted hepatocytes viability. Around 70% of hepatocytes were lost early after transplantation due to neutrophil and macrophage/monocyte infiltration elicited by complement activation and cytokine release (Gupta et al., 1999b). Therefore, small molecules targeting these aspects are effective in improving hepatocyte engraftment.

Complement system initializes innate immune system activation and shares a crosstalk with coagulation system. Direct inhibition of complement system hinders the activation of innate immune system and thus enhances cell engraftment. Kusakabe J et al. reported that PMX53, a C5a receptor 1 (C5aR-1) antagonist, mitigated hepatic IRI through inhibiting pro-inflammatory cytokine release and neutrophil/macrophage infiltration and platelet aggregation (Kusakabe et al., 2020), suggesting the crosstalk between coagulation and complement system. The C5aR inhibitor may also exert effect in hepatocyte induced IBMIR due to similar process with IRI. Noteworthy, our lab demonstrated that a clinically-used ROCK inhibitor, ripasudil, significantly enhanced hepatocyte engraftment and liver repopulation by blocking membrane attack complex (MAC) formation *in vitro*/vivo and thereby inhibiting KCs functions (Ma et al., 2023). In isolated hepatocytes, the endocytosis of cell membrane protein, including CD59a, was observed (Ma et al., 2023). Mechanistically, ripasudil safeguarded membrane localization of CD59a, a MAC inhibitor highly expressed on hepatocytes, hindering the MAC formation (Ma et al., 2023). In addition, ripasudil improved liver repopulation (3.96% *versus* 0.52%) and increased alanine aminotransferase and aspartate aminotransferase levels at 4 weeks post-transplantation, suggesting that ripasudil exerted long-term effect on engrafted hepatocytes and liver function. Therefore, ROCK inhibitor ripasudil is a promising chemical to inhibit complement activation and thus promote hepatocyte engraftment.

Innate immune cells are the most important scavengers responsible for hepatocyte clearance in the short period after HTx. Thus, investigators have developed various strategies to inhibit innate immune cells for improving hepatocyte engraftment. Gadolinium chloride (GdCl_3_), a lanthanide compound, depletes KCs irreversibly and has been widely used in research. Joseph B et al. indicated that depleting KCs with GdCl_3_ increased the number of transplanted hepatocytes in short and long-term after HTx, resulting in significantly improved liver repopulation (Joseph et al., 2002). However, GdCl_3_ permanently destroys KCs by blocking K-type calcium channels (Pałasz and Czekaj, 2000), thus unsuitable for clinical use. Transcription factor GATA-1 competed with c-Jun for the β3/β4 region of PU.1 protein, thereby preferring erythropoiesis and suppressing myelopoiesis (Raghav and Gangenahalli, 2021). Activation of GATA-1 by small molecules may decrease Kupffer cell number in the liver and improve hepatocyte engraftment. Glycine, a nonessential amino acid, has also been used to inhibit KC function in liver without decreasing KC numbers (Rentsch et al., 2005). After human adipose-derived stem cells transplantation, the number of KCs and TNFα release markedly increased (Al-Saeedi et al., 2019). Glycine treatment did not decrease KC numbers but significantly downregulated TNFα levels, resulting in improved cell engraftment in liver (Al-Saeedi et al., 2019).

In addition to direct KC inhibitors, several small molecules mentioned above impaired KC fuctions and enhanced cell engraftment. Darusentan treatment significantly declined KC numbers likely by downregulating macrophage chemokines Ccl2 and Cx3cL (Bahde et al., 2014). Thalidomide decreased KC-associated inflammatory cytokines/chemokines expression and infiltrated KCs (Viswanathan et al., 2016), probably via inhibiting NF-kB signaling and TNFα (Mercurio et al., 2017). In hepatic IRI model, neutrophil infiltration and TNF-α release caused by IRI was impaired by dabigatran (Noguchi et al., 2021). Thrombin, also known as factor II, upregulates proinflammatory cytokine release and promotes leukocyte infiltration (Esmon, 2014). Therefore, inhibition of thrombin by dabigatran may impair the IRI-induced inflammatory response. The dual or multiple anti-IBMIR functions of these small molecules indicate close crosstalk between coagulation, ischemia, and inflammation. Simultaneous targeting of these processes by single molecule is the future direction of improving cell engraftment.

### 4.4 Promote the proliferation of transplanted cells

In Fah^−/−^ mice, donor hepatocytes have a proliferative advantage for liver repopulation since host cell proliferation is impaired. (Overturf et al., 1996). Our lab demonstrated that host hepatocytes in Fah^−/−^ mice liver experienced senescence characterized by cell cycle arrest, which favored the proliferation of donor hepatocytes (Chen et al., 2019). Senescence disrupts cell connections and degrades extracellular matrix, providing engraftment space (Chen et al., 2019). Among patients with Crigler–Najjar disease where the proliferation of host hepatocytes is impaired, transplanted hepatocytes successfully alleviated the liver functions (Jorns et al., 2012). Moreover, in α1-antitrypsin deficiency mice models with inhibited host hepatocytes proliferation, wild-type donor hepatocytes replaced 20%–98% of host hepatocytes (Ding et al., 2011). However, in most inherited metabolic disorders host hepatocytes viability is not significantly compromised (Barahman et al., 2019a). The proliferation advantages can be achieved by: (1) inhibit host cells proliferation and (2) promote transplanted cells proliferation. For the former one, several precondition strategies including hepatic irradiation, portal embolization, and partial hepatectomy are used in clinical trials (Nguyen et al., 2020). However, these methods are invasive to patients’ body. Using small molecules to intervene with host and donor hepatocytes viability is an ideal, noninvasive, and controllable method.

The Hippo pathway, critical for liver regeneration, comprised of MST1/2 phosphorylating and activating the LATS1/2, which then phosphorylates and inactivates YAP and TAZ. Deletion of MST1/2 or LATS1/2 genes increased nuclear level of YAP/TAZ and expression of downstream genes (Driskill and Pan, 2021). Fan F et al. identified a MST1/2 inhibitor, XMU-MP-1, effectively suppressed MST1/2 kinase and thereby activated downstream YAP in various cells. In Fah^−/−^Rag2^−/−^IL2rg^−/−^(FRG) mice treated with XMU-MP-1, transplanted hepatocyte proliferation was remarkably promoted, improving liver repopulation and ALB levels (Fan et al., 2016).

HGF/c-Met signaling pathway plays an important role in liver regeneration. HGF binds to its specific receptor c-Met, activating downstream pathways and leading to cell proliferation (Zhao et al., 2022). It was reported that the c-Met receptor agonist antibody 5D5 induced significant proliferation in hiPSC-derived hepatocyte-like cells (hiPSC-HLCs) *in vitro* (Yuan et al., 2019). In FRG-SCID mice, 5D5 remarkably promoted liver repopulation rate of hiPSC-HLCs(Yuan et al., 2019). Small molecule agonists targeting c-Met receptor warrant further development.

Jiang M et al. observed that the HPC and cell cycle genes significantly increased from day 4 after HTx in Fah^−/−^ mice, peaked at day 30 and then decreased to normal. The labeled hepatocytes expanded at day 30 and almost repopulated the liver at D120 (Jiang et al., 2023). These results suggested that the transplanted hepatocytes dedifferentiate to an HPC stage to proliferate and then re-differentiate to hepatocyte after repopulation. Combination of ROCK inhibitor Y27632 and CHIR99021 (termed YC) markedly increased the expression of HPC and cell cycle genes *in vivo* and thereby promoted hepatocytes proliferation in treated mice (Jiang et al., 2023). Clinically used small molecule drugs of ROCK signaling Netarsudil (N) and Wnt signaling LY2090314 (L) were also tested. Similar to YC, NL treatment also promoted the dediffereantiation and redifferentiation of hepatocytes and favored the liver repopulation *in vivo* (Jiang et al., 2023).

## 5 Conclusion and future perspectives

Hepatocyte transplantation (HTx) provides an alternative way of liver transplantation for patients with irreversible liver diseases. Translation of HTx from lab to bedside is of great emergency due to the scarcity of donor livers. However, the wide application of HTx is hindered by two main problems: shortage of high-quality donor hepatocytes and low cell engraftment and repopulation rate.

### 5.1 Cell source for HTx

Chemical induction of hepatic fate from pluripotent stem cells (PSCs) can provide an unlimited hepatocyte source. The insights into the signaling pathway and transcription factor network of liver development provide cues for yielding hepatocyte-like cells (HLCs) from PSCs by chemical modulation. During embryonic liver development, bone morphogenic protein (BMP), Wnt/β-catenin and Notching signaling promote definitive endoderm (DE) formation; fibroblast growth factors (FGFs) and BMPs promote hepatoblasts (HBs) specification from DE; Notch and TGFβ signaling inhibition and Hippo signaling activation drive HBs into hepatocyte fate. Downstream of signaling pathways, transcription factors including FOXAs, GATA4/6, HNF4α, and HNF1 interact with each other and activate liver-specific gene expression, accompanied by epigenetic changes. For instance, pioneer factors recruit the histone acetyltransferase p300 to deposit H3K9ac/14ac at liver-specific gene regulatory elements, directing hepatic programs (Xu et al., 2011). To recapitulate liver development, the differentiation of PCSs comprises three main steps: ‘PSCs to DE’, ‘DE to hepatic progenitor cells (HPCs)’, and ‘HPCs to HLCs’. Stepwise chemical approaches derive HLCs by regulating these developmental paradigms and epigenetic landscapes. Additionally, reopening liver genes with small molecules enables direct fibroblast reprogramming (Rombaut et al., 2021; Park et al., 2019; Bai et al., 2023).

A comparative study showed that the chemically induced hepatocytes resemble growth-factor derived counterparts (Gao et al., 2020). The expression of liver drug-metabolizing enzymes, transporters, and nuclear receptors in primary hepatocytes are significantly higher than that in HLCs (Gao et al., 2020).

Despite the progress made in chemical induction of hepatocyte, it is noteworthy to mention that consensus on optimal small molecule combinations, doses and hepatocyte quality is lacking, hindering protocol standardization and clinical translation. Future work establishing current good manufacturing practice (cGMP)-grade protocols and improving functional stability is needed for transplantation applications. Comparing differential gene expression between primary and induced hepatocytes may identify targets to enhance cell competence for transplantation.

### 5.2 Strategies to improve low cell engraftment

In general, there are two strategies to improve low cell engraftment: prime the host hepatic microenvironment for HTx (hepatic irradiation and partial hepatectomy) and prime the donor hepatocytes (alginate encapsulation) (Nguyen et al., 2020). Hepatic irradiation (HIR) has manifested its efficiency in animal preclinical studies and clinical trials (Soltys et al., 2017; Barahman et al., 2019b). However, the efficiency depends on irradiation parameters and host radiosensitivity. It is noteworthy that HIR has hepatoxicity and may cause side effects in patients. Therefore, combination of HIR and systemic small molecules including anti-IBMIR and chemotherapeutic drugs may reduce dosage of both radiation and small molecules, lowering the risks of adverse drug reactions. Recent studies have shown that hepatocyte microbeads encapsuled by alginate are protected from immune rejection and improve cell engraftment (Jitraruch et al., 2014). A recent clinical trial has validated its efficiency and safety in pediatric patients with ALF (Dhawan et al., 2020). However, other problems involved in HTx such as microcirculatory obstruction cannot be tackled with alginate encapsulation. It is interesting to envisage that load alginate encapsulation with small molecules can improve cell engraftment.

Challenges to chemical approaches still remains to be solved before wide applications, including drug specificity, target accuracy, pharmacokinetic properties, and safety. For instance, CHIR99021, a GSK3β inhibitor that activates Wnt/β-catenin signaling, is widely used in regulating cell differentiation, reprogramming, and proliferation. However, Wnt/β-catenin signaling activation promotes the development of liver cancer and the resistance to immunotherapy (Zhou et al., 2022; He and Tang, 2020). Hippo pathway play critical role in liver physiology and tumorigenesis. Depletion of MST1/2 drives the formation of hepatocellular carcinoma and/or cholangiocarcinoma (Driskill and Pan, 2021). Whether *in vivo* administration of Wnt/β-catenin (CHIR990221) and Hippo signaling activators (XMU-MP-1) will lead to tumorigenesis or affect tumor development in chronic liver failure recipients with hepatocellular carcinoma needs to be studied. TGFβ signaling, ROCK signaling, and others are ubiquitous and regulates numerous critical physiologic processes in healthy cells. How to minimize the systemic effect while maximize the hepatic effect of *in vivo* administration of small molecules is an important problem before the large-scale clinical use. Developing liver-specific or hepatocyte-specific drug delivery system is one of the feasible strategies, including nanoparticle-based and small molecule-based drug delivery systems (Unagolla et al., 2024; Sharma et al., 2021). Off-target effects are another challenge of clinical application of small molecules. *In vitro* screening is insufficient to mimic the *in vivo* complicated microenvironment. Therefore, small molecules may cause off-target effects *in vivo* despite its *in vitro* efficacy. Target prediction models based on deep learning are prosperous strategies to mitigate off-target effects.

Despite the obstacles, some small molecules mentioned in this review have already applied clinically, whose application in HTx can be quickly translated due to its confirmative safety and efficiency in human (Table 3). For instance, ripasudil has already been licensed for treating ocular hypertension and open-angle glaucoma, which has potential for rapid translation as a therapeutic to promote hepatocyte engraftment (Table 3). It is noteworthy that systemic adverse drug reactions need further investigation if ripasudil is used systemically, as it rarely enters systemic circulation through the blood-ocular barrier when administered as eye drops in current clinical trials (Kusuhara and Nakamura, 2020). The indication and clinical trials of other small molecules in this review was accessible in Table 1 and 2. In the future, studies comparing various chemical protocols of hepatocyte differentiation and reprogramming are urgently needed in order to standardize a paradigm protocol for potential clinical trials. Additionally, studies on simultaneous interventions of each process during hepatocyte engraftment are warranted, because the reason of low cell engraftment is multifactorial. We believe that the in-depth probes into stem cell biology and progress in chemical screenings and deliver systems will promote the application of HTx by ingenious chemical formula in the future.

**TABLE 3:** Small molecules undergoing clinical trials and their safety outcomes.

Small molecule	Clinical trial	Condition	Administration and dose	Adverse event	FDA approval and indication
Sodium butyrate	NCT04415333	Hypertension	80 mmol in a 0.9% saline solution, via rectum	no AE	-
	NCT05456763	IBD	150 mg PO QD	no AE	
Valproic acid	NCT06248931	Migraine	400 mg PO QD	22 patients (78.6%) reported mild or moderate AE, including increased appetite, hair loss, somnolence, etc.	Approved for seizure disorders, mania, and prophylactic treatment of migraine headache
	NCT04698525	Migraine prophylaxis	500 mg PO BID	7 patients (41.18%) reported mild or moderate AE, including somnolence and parasomnia	
	NCT03885154	Pediatric migraine	20 mg/kg IVGTT QD, followed by 1 mg/kg/h IVGTT for 24 h	no AE	
Dimethyl sulfoxide	NCT05223244	Interstitial cystitis	50 mL of DMSO with 1 mL of triamcinolone (10 mg/mL), bladder instillations	no AE	Approved for the symptomatic relief of interstitial cystitis
Parnate	NCT01430455	Bipolar depression	10–120 mg PO QD	6 (85.71%) patients reported mild or moderate AE	Approved for major depressive episode without melancholia
	NCT02273102	Refractory AML and MDS	20 mg PO BID	The most common AE of all grades were fatigue, creatinine increased, dizziness, dry mouth, headache, rash The most common serious AE were febrile neutropenia and pneumonia	
Darusentan	NCT00330369	Hypertension	50mg, 100mg, 300 mg PO QD	The most common AE is edema and/or fluid retention 57 (70.37%) patients reported mild or moderate AE and 1 (1%) patient had NSTEMI in 50 mg group 60 (74%) patients reported mild or moderate AE, 2 (3%) patient had serious AE (NSTEMI and atrial fibrillation) in 100 mg group 63 (74.11%) patients reported mild or moderate AE, 2 (2%) patients had serious AE (fluid retention and heart failure) in 300 mg group	-
	NCT00389779		50mg, 100mg, 300 mg PO QD	The most common AE is edema and/or fluid retention 266 (73%) patients reported mild or moderate AE 23 (6%) patients had serious AE (death, liver function test abnormality, cardiac-related serious AE, and allergy-related serious AE)	
Ripasudil	NCT04620135	Primary open-angle glaucoma	0.4% ophthalmic solution BID	2 (1.63%) patients had serious AE (peumonia and erythema multiforme)	-

Abbreviation: PO QD, administered orally one time daily; PO BID, administered orally twicw daily; IVGGT, intravenous drip; AML, acute myeloid leukemia; MDS, myelodysplasia; IBD, inflammatory bowel disease; NSTEMI, Non-ST, segment elevation myocardial infarction; AE, adverse event.

## Glossary

HTx: hepatocyte transplantation
OLT: orthotopic liver transplantation
ALF: acute liver failure
PHHs: primary human hepatocyte
HLC: hepatocyte-like cells
IBMIR: instant blood-mediated inflammatory reaction
BMP: bone morphogenic protein
DE: definitive endoderm
PFG: posterior foregut
HB: hepatoblast
FGFs: fibroblast growth factors
LSD1: lysine-specific demethylase 1
PSCs: pluripotent stem cells
hESCs: human embryonic stem cells
CHIR: CHIR99021
AFP: fetoprotein
ALB: albumin
PROX1: prospero homeobox 1
NaB: sodium butyrate
DMSO: dimethyl sulfoxide
HGF: hepatocyte growth factor
HPCs: hepatic progenitor cells
FH1: functional hits 1
FPH1: functional proliferation hit 1
cGMP: current good manufacturing practice
hiPSC: human iPSC
MEFs: mouse embryonic fibroblasts
iHeps: induced hepatocyte-like cells
VPA: valproic acid
XEN: extra-embryonic endoderm
KMTs: histone methyltransferases
DNMTs: DNA methyltransferases
HDAC: histone deacetylase
KCs: Kupffer cells
HSCs: hepatic stellate cells
TF: tissue factor
MAC: membrane attack complex
GdCl3: Gadolinium chloride
HIR: hepatic irradiation
